# Exploring Geographic Variation of Mental Health Risk and Service Utilization of Doctors and Hospitals in Toronto: A Shared Component Spatial Modeling Approach

**DOI:** 10.3390/ijerph15040593

**Published:** 2018-03-26

**Authors:** Jane Law, Christopher Perlman

**Affiliations:** 1 School of Planning, University of Waterloo, Waterloo, ON N2L 3G1, Canada; 2 School of Public Health and Health Systems, University of Waterloo, Waterloo, ON N2L 3G1, Canada; chris.perlman@uwaterloo.ca

**Keywords:** health inequality, hospital admissions, physicians, Bayesian spatial analysis, geographic information systems (GIS), health services, shared component models

## Abstract

Mental Health has been known to vary geographically. Different rates of utilization of mental health services in local areas reflect geographic variation of mental health and complexity of health care. Variations and inequalities in how the health care system addresses risks are two critical issues for addressing population mental health. This study examines these issues by analyzing the utilization of mental health services in Toronto at the neighbourhood level. We adopted a shared component spatial modeling approach that allows simultaneous analysis of two main health service utilizations: doctor visits and hospitalizations related to mental health conditions. Our results reflect a geographic variation of both types of mental health service utilization across neighbourhoods in Toronto. We identified hot and cold spots of mental health risks that are common to both or specific to only one type of health service utilization. Based on the evidence found, we discuss intervention strategies, focusing on the hotspots and provision of health services about doctors and hospitals, to improve mental health for the neighbourhoods. Limitations of the study and further research directions are also discussed.

## 1. Introduction

The availability of and access to health services provides an indication of the responsiveness of health systems to the population needs. According to the behavioral model of health service use, access to or utilization of health services may result from predisposing, enabling, and need factors that exist at individual and contextual levels [1,2,3]. Anderson et al. [3] describe predisposing factors to include the policy environment at the contextual level and age or sex at the individual level; enabling contextual factors include the availability and supply of health services while individual enabling factors include health insurance or having a primary care physician; needs could include the prevalence of mental health conditions within a population at the level of context and the presence of mental health symptoms experienced by an individual. Within mental healthcare, such a framework can be used as a basis for exploring the equitable distribution of health service use, including use of advantageous (e.g., primary care) and adverse (e.g., hospitalization) services.

### 1.1. The Mental Health (MH) System in Ontario, Canada 

There are emerging calls for health systems to be more responsive to the prevention, early identification, and management of mental health conditions. Primary health care services can play an important role for supporting the early identification and management of MH conditions, but do we have a sufficient and accessible supply of primary health care services nationally, provincially, and more importantly locally? Canada has been found to have the lowest number of physicians available to the population and higher occupancy rates for acute care beds in hospitals compared to the UK, U.S., and Sweden in 2005 [4]. The country has a universal health system where provincial governments pay for most hospital and primary care physician services. In Canada, primary care physicians work on fee-for-service or various forms of capitation-based funding. These models may influence physician practices, such as rostering of mental health clients [5].

In Ontario, mental healthcare often includes counselling services, community-based services offered through organizations such as the Canadian MH Association, and hospital services [6]. In many instances, the person may not have or may seek out a primary physician for a MH condition. For this reason, some health regions have established telehealth support lines, such as ConnexOntario (ConnexOntario is a Canadian health services information organization that is funded by the Ontario Ministry of Health and Long Term Care. The organization provides helplines, online resources, reports, and data related to the availability of mental health, substance use, and problem gambling services in Ontario. (www.connexontario.ca)) or here247.ca (Here247 is a telephone and online resource for mental health, addictions, and crisis support to the Waterloo-Wellington Local Health Integration Network (LHIN), one of 14 health regions in Ontario, Canada. This program receives public funding from the LHIN) to support screening and referral to service. Individuals experiencing a crisis, such as symptoms of psychosis and self-harm ideation, may also end up in hospital emergency departments or acute psychiatric care settings. In other instances, a planned hospital admission may occur for specialized care for persons experiencing severe and chronic MH conditions. After discharge from hospital, best practice dictates that a physician in the community follows up with the person within 7 to 30 days, although research has shown that this rarely happens [7]. Optimally, sufficient supply of community-based MH services that include primary care physicians should reduce the need for hospital-based care. 

Both primary health care and hospital services play an important role in population health; however, it has been a challenge to supply and balance these services satisfactorily worldwide. In Ontario, individuals with MH conditions utilize more primary care, emergency department, and hospital services than persons with non-MH conditions for both MH and non-MH reasons [8]. The first point of healthcare contact for persons with MH conditions is often the hospital. For instance, Gill and colleagues (2016) found that over half of children and youth in Ontario, Canada who accessed an emergency department for MH reasons had no prior contact with outpatient MH services, and that the rates of first contact in the emergency department were highest among those with no primary care physician [9]. This trend is concerning given that contact with a physician or psychiatrist before hospitalization is strongly related to receiving appropriate follow-up care following a first-episode psychosis [10]. Follow-up within 30-days of discharge from hospital for persons with schizophrenia is associated with reduced risk of readmission within 30-days [7]. These patterns underscore the importance of having access to outpatient physician care among persons with MH conditions. 

Local neighbourhoods with a lack of physicians could lead to more needs for hospital services. Where are the neighbourhoods displaying high or low risk of MH visits to physicians (referred to as doctors below in accordance with our source of data) or hospitals? Are there neighbourhoods displaying high risk of MH visits to doctors but low risk of visits to hospitals and vice versa? If so, where are these neighbourhoods? Addressing these questions could provide insights into the use, supply, and needs of physicians versus hospitals at the area level for intervention at specific local areas to improve population health.

### 1.2. Exploring Geographic Variation

Spatial analysis has been commonly used to explore geographic patterns of mental disorders. For instance, a number of hotspots of the prevalence of treated mental illness were identified in Barcelona using Multi-Objective Evolutionary Algorithm [11]. Ngui and Vanasse [12] demonstrated the use of spatial techniques to identify clustering of MH service locations across areas within the southwest region of Montreal. Such techniques may be important for identifying disparities or variations in service availability and use. However, the majority of the existing research on geographic patterns or distributions of MH analyzed a single outcome, such as hospital admissions or general health service utilization in one model. They do not simultaneously account for MH incidence or prevalence by looking at visits to doctors and hospitals within one model. 

This study explores common and discrepant geographic pattern of MH from doctor visits (DV) and hospital admissions (HA) using one (joint) model. The model is useful for identifying their similarities and dissimilarities in geographical distributions, informing risks of MH at the neighbourhood level, and reflecting the demand of health services. Results of the study could provide a means of allocating public health services in MH.

The model used in this study adopts a shared component spatial modeling (SCSM) approach that applies Bayesian spatial statistics [13,14]. It jointly analyzed the geographic variation of two indicators of MH service utilization in the population from two sources: counts of visits to doctors and hospital admissions. Data of MH risk in each neighbourhood from either source may involve issues of underreporting caused by detection bias and neighbourhood misclassification caused by geocoding errors, for instance. Bayesian spatial analysis enables the borrowing of strength from neighbours and thus could provide more precise estimates of risk of MH in each neighbourhood compared to its frequentist counterpart. Its applications have focused almost exclusively on spatial modeling of a single outcome or source. However, when there are similar patterns of geographic variation of two or more related outcomes, then a joint analysis could provide more convincing estimates of the underlying risk surface. Joint analysis of multiple outcomes by SCSM that is an extension of the most frequently used Besag, York and Mollie model (BYM) [15] has been found to perform better than separate analyses of individual outcomes by the BYM [16].

**Figure 1:**
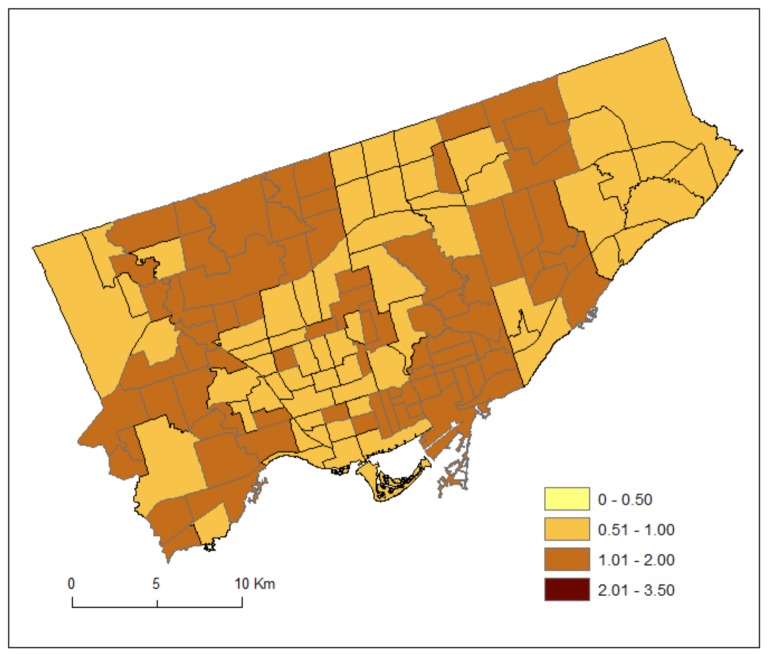
Standardized prevalence ratios of mental health conditions by Toronto neighbourhoods for doctor visit (DV) (2011–2012).

**Figure 2:**
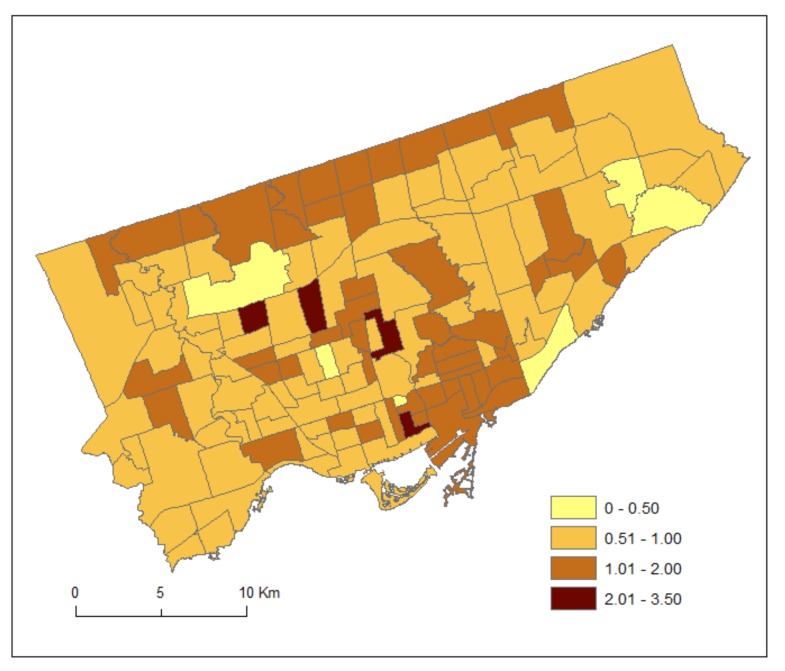
Standardized prevalence ratios of mental health conditions by Toronto neighbourhoods for hospital admissions (HA) (2012–2013).

The use and analysis of more than one data sources (DV and HA) with SCSM helps to increase statistical power as the model could strengthen inference and correct for any spatially structured sources of bias [17]. The model jointly analyses DV and HA while accounting for spatial autocorrelation (i.e., similar neighboring values), which is apparent from the prevalence maps of DV and HA related to mental health (Figure 1 and Figure 2). The shared component can be viewed as a weighted average of spatial random effects from individual (separate) models of DV and HA. Results provide estimates of a risk surface common to both suggesting common risk factors, a risk surface specific for DV, and a risk surface specific for HA. This may provide more convincing evidence of real clustering in the underlying risk surface of MH and help to explore if there are different geographical patterns between DV and HA, and thus possibly different features of the two data sources about MH service utilization. Findings may suggest that hospitalizations could be preventable with timely and good primary care from DV for areas that have a high risk of HA but not DV. They may also flag those areas that have MH issues but relatively good provisions of doctors resulting in high risk of DV but not HA. Further, both services of HA and DV may be showing a deficiency for areas that are identified as having both a high risk of DV and HA.

Section 2 describes the study region, data, and shared component spatial model. Section 3 presents the results. Section 4 discusses the results, methods, and data. Section 5 is the conclusions with recommendations for future research.

## 2. Materials and Methods

### 2.1. Study Region and Data

The City of Toronto contains 140 neighbourhoods that were aggregated from census tracts and created by the Social Policy Analysis and Research unit in the City’s Social Development and Administration Division with assistance from Toronto Public Health. The average population of a neighbourhood is 7000 to 10,000 [18]. These neighbourhoods have also been referred to as social planning neighbourhoods [19]. 

This study analyzed MH service use among persons aged 20+ at the neighbourhood level in the city of Toronto from visits to doctors (DV) and hospital admissions (HA). The data were based on the residence of individuals. All neighbourhoods contain at least five counts. The data for DV contains (1) the average number of patients ages 20+, both sexes, by Toronto neighbourhoods with (one or more) Ontario Health Insurance Plan (OHIP) MH billing code during the 2011 and 2012 fiscal years (1 April 2011 to 31 March 2012); and (2) the population aged 20+ who were alive and living in the City of Toronto on 1 April, 2012 from the Ontario Ministry of Health and Long-Term Care Registered Persons Database. Similarly, the data for HA contains all unscheduled Hospital admissions ages 20+, both sexes, by Toronto neighbourhoods for MH Conditions for 2012–2013 and 2013–2014 (1 April 2012–31 March 2014) observation period with the same population data as above. Data for HA were based on the Ontario MH Reporting System (OMHRS), a mandated system that includes records of all admissions to adult designated inpatient MH beds, including beds in general community hospitals, provincial psychiatric, and specialty psychiatric facilities [20]. It is important to note the differences in the two data sources in measuring MH service use. For DV, MH conditions are defined by the occurrence of a doctor’s visit for a symptom related to MH [21]. For HA, the data capture hospitalizations in adult designated inpatient MH beds, including beds in general, provincial psychiatric, and specialty psychiatric facilities [22]. 

For each neighbourhood, we divided the counts by two to estimate the average number of patients during the 2012 and 2013 fiscal years (1 April 2012–31 March 2013). It is acknowledged that the estimated counts are inaccurate as they are based on the 2011 Census population estimates [23]. Figure 1 and Figure 2 shows the standardized prevalence ratios of MH by Toronto neighbourhoods for DV (2011–2012) and HA (2012–2013), respectively.

### 2.2. Shared Component Spatial Modeling (SCSM)

We adopted the approach of SCSM proposed by Held et al. [14] to combine outcomes, types of MH service use, from the data sources of DV and HA. The method of SCSM, which is an extension of the BYM that contains spatially-structured and unstructured random effects, has been shown to improve inference over separate analysis of each outcome [17,24,25]. 

We assume that the likelihood for MH from counts of DV and HA in the ith area, O_i1_ and O_i2_, respectively, is independent Poisson, conditional on an unknown mean of λ_i1_ and λ_i2_, respectively. For this study, i = 1, 2, …, n, where n = 140, the total number of neighbourhoods or areas. The assumption of conditional independence is valid given knowledge of the model parameters when correlation is accounted for within the model by spatial random effects, which we described below. A binomial distribution can be used instead if counts are not low.

O_i1_ ~ Poisson (λ_i1_), (1)

O_i2_ ~ Poisson (λ_i2_), (2)

λ_i1_ = e_i1_r_1i_, (3)

where e_i1_ and r_1i_ are the expected count and relative risk for area i for DV, respectively.

λ_i2_ = e_i2_r_2i_, (4)

where e_i2_ and r_2i_ are the expected count and relative risk for area i for HA, respectively.

The expected count of DV or HA for each area is the overall rate of DV or HA in Toronto multiplied by the corresponding denominator (population) described in the data section above.

The main assumption is that DV and HA share a similar spatially structured pattern of risk describing the true underlying risk surface of MH, as modeled below.

log (λ_i1_|**μ**) = log (e_i1_) + α_1_ + δθ_i_ + s_1i_ + u_1i_, (5)

log (λ_i2_|**μ**) = log (e_i2_) + α_2_ + (1/δ)θ_i_ + s_2i_ + u_2i_, (6)

where λ_ik_|**μ** is the expectation of O_ik_ (k = 1, 2; k = 1 for DV, k = 2 for HA) conditioning on the random effects **μ** = (**θ^T^**, **s**_1_**^T^**, **s**_2_**^T^**, **u**_1_^T^, **u**_2_^T^)^T^, **θ** = (θ_1_, θ_2_, … , θ_n_)^T^ denotes a random effects ensemble representing shared risk effects that are common to both DV and HA, **s**_k_ = (s_k1_, s_k2_, …, s_kn_)^T^ denotes a spatial random effects ensemble that only has relevance to the kth outcome, **u**_k_ = (u_k1_, u_k2_, …, u_kn_)^T^ denotes an unstructured random effects ensemble that only has relevance to the kth outcome.

So,

r_1i_ = exp(α_1_ + δθ_i_ + s_1i_ + u_1i_), and (7)

r_2i_ = exp(α_2_ + (1/δ)θ_i_ + s_2i_ + u_2i_), (8)

α_1_ and α_2_ are specific intercepts for DV and HA, respectively, denoting DV/HA specific baseline risks. The global mean rate of DV and HA are exp(α_1_) and exp(α_2_), respectively. We assigned flat normal priors to α_1_ and α_2_. The (separate) random component **u_1_** is only relevant to DV. It allows for non-spatially-structured differences between the risk surfaces from DV and HA, similarly for **u_2_** that is only relevant to HA. We assigned normal priors to **u_1_** with a mean of zero and a precision parameter. Similarly, for **u_2_**. The **s_1_** and **s_2_** terms denote residual spatially structured random effects specific to DV and HA, respectively. Residual data source specific variations may indicate some spatially structured dissimilarities of risk surfaces due, for instance, to biases in the collection of data for DV and HA.

The **θ** term denotes the latent (shared) component of the spatial pattern of risks shared by both DV and HA. It estimates the relative weight of each data source (DV or HA) for MH service use. It can be considered as a surrogate for unobserved factors that may explain the geographical variations of the risk of MH from both DV and HA. An area (i) with a high risk of both DV and HA is indicated by a large positive value of θ_i_. 

The contribution of the shared component **θ** to MH risks was weighted by the scaling parameter δ (δ > 0). This scaling parameter (δ) is necessary for identifiability [26]. It allows different risk gradients for the shared component due to differences in the relative sensitivity of DV and HA to classify MH. Following Knorr-Held and Best [13], we assumed that the logarithm of δ has a normal prior with mean 0 and precision 5.9 (=1/0.17), which means a prior median for δ equals 1 and a prior belief that the ratio of the risk gradients (δ and 1/δ) is between 1/5 and 5 with 95% probability.

The three components, **θ**, **s_1_** and **s_2_**, were assumed to be independent, with each one following an intrinsic conditional autoregressive spatial prior distribution that assumes that parameter values in adjacent neighbourhoods are similar [15,27]. The conditional distribution of θ_i_ given θ_j_ is normal with mean $\bar{\theta_{i}}=\underset{j\neq i}{\sum }\omega_{i,j}s_{j}/m_{i}$ and variance = $\omega_{\theta }^{2}/m_{i}$. $\left\{\omega_{i,j}:i,j=1,\;2,\;\ldots ,n\right\}$ is a 0–1 contiguity matrix (**W**) in which ω_i,j_ equals one if i and j are neighbours and zero otherwise, and ω_i,i_ equals 0. $m_{i}=\underset{j}{\sum }\omega_{i,j}$, is the number of neighbours of area i. Similarly, for the conditional distribution of **s_1_** and **s_2_**. The spatial prior enables estimation of additional information on MH through borrowing strength across the data sources as well across neighbouring areas. The three components act as surrogates of (unobserved) covariates that have substantial spatial structure with different spatial patterns. The model assumes that there are no interactions between the (true) covariates. Despite the assumption, this model that contains no actual covariates could provide a useful approximation to the underlying risk surface and insight into the pattern of exposure that is relevant to each of outcomes being analyzed [13]. However, identifiability could be an issue when the data do not suggest evidence of shared (risk) factors that is assumed by the model [25]. Mapping the outcomes to explore if common geographic patterns and shared factors may exist before modelling the multiple outcomes could be helpful.

Having the same specific components for both outcome variables of DV and HA allows a symmetric formulation but could cause problems with the identifiability of the latent spatial fields when there is no strong prior assumption on the precision parameter [14]. Following Held et al. [14], we used a weakly informative hyperprior of Gamma (1.0, 0.01) for all the precision parameters as a non-informative choice may lead to an improper posterior distribution. The hyperprior represents a prior mode at 0.005 and infinite expectation and variance. For sensitivity test, we used different hyperpriors for the precision parameters: Gamma (0.1, 0.1) for all the spatially-structured random effects, and Gamma (0.01, 0.01) for all the unstructured random effects [17]. The Deviance Information Criterion (DIC) was used to compare model fit. In simple terms, smaller values of DIC indicate better fitting models [28].

The models were fitted using Markov chain Monte Carlo simulations via the WinBUGS software (The BUGS Project: Cambridge, London) with two parallel chains. Convergence was monitored by trace plots, autocorrelation graphs, and the Gelman–Rubin convergence statistic [27]. Results from WinBUGS were exported to the shapefile of Toronto neighbourhoods, which can be mapped using most mapping software.

## 3. Results

The models that applied different hyperpriors of precision parameters for sensitivity tests gave similar results. According to the DICs, the model that applied the hyperpriors proposed by Held et al. [14] fitted the data better than the sensitivity-test model that applied the hyperpriors proposed by Ancelet et al. [17]. The DIC difference was 16. We reported below the results of Held et al.’s model that were based on 40,000 (20,000 from each chain) samples with a thinning of 100 after convergence of the two chains by 20,000 iterations. The model fits the data well with good convergence and no identifiability issues, as reflected by the trace plots (random scatter about a stable mean value), autocorrelation functions (high autocorrelation graph, near to one), and Gelman–Rubin convergence statistic (converge to one). For all of the estimated parameters, the Monte Carlo error was less than five percent of the sample posterior standard deviation of the samples taken [27]. 

**Figure 3:**
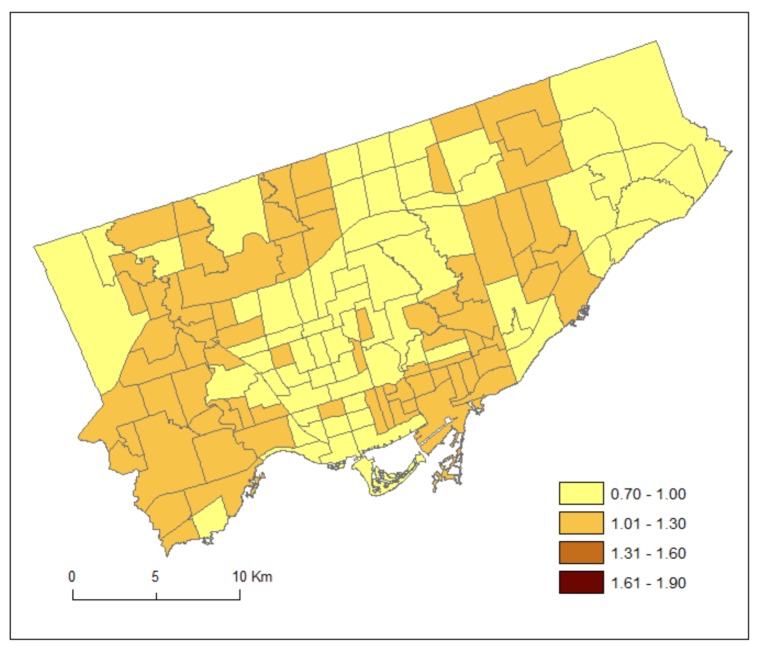
Posterior medians of the risk patterns of the area-specific risks for DV, r_1i_.

**Figure 4:**
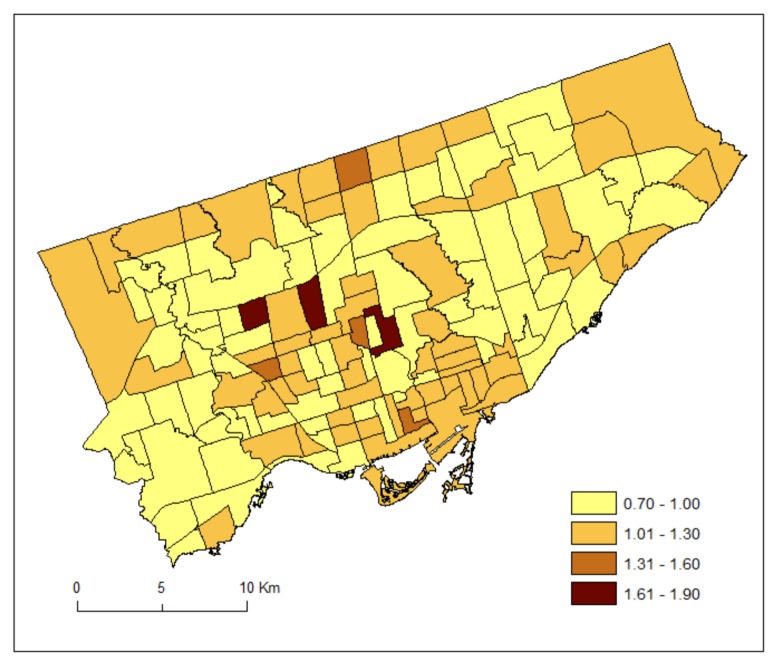
Posterior medians of the risk patterns of the area-specific risks for HA, r_2i_.

Figure 3 maps the posterior medians of the risk patterns of the area-specific risks for DV, r_1i_. It shows area-specific risks and hotspots of DV. Figure 4 maps the posterior medians of the risk patterns of the area-specific risks for HA, r_2i_. It shows area-specific risks and hotspots of HA. 

**Figure 5:**
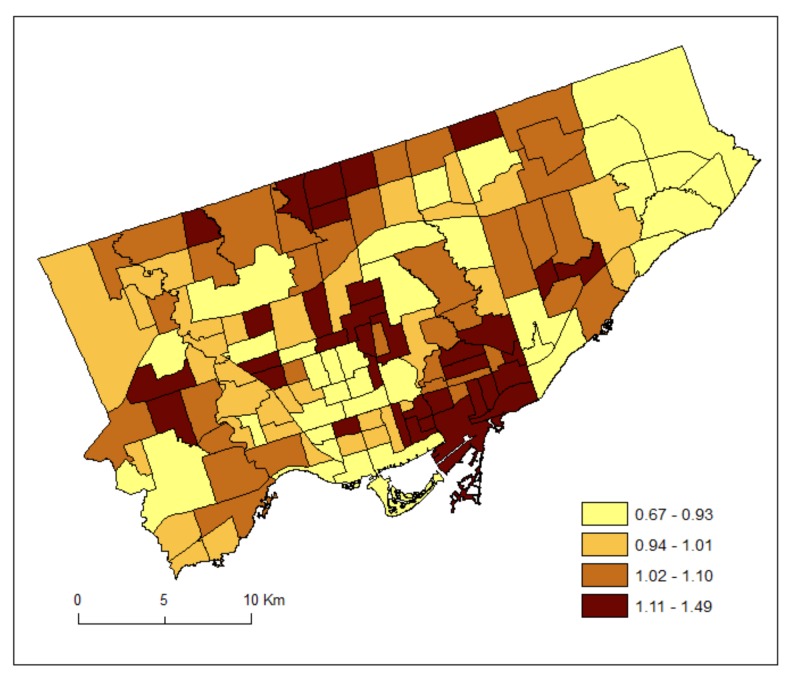
A quantile map of the true risk surface of MH service use from the posterior medians of exp(θ_i_).

**Figure 6:**
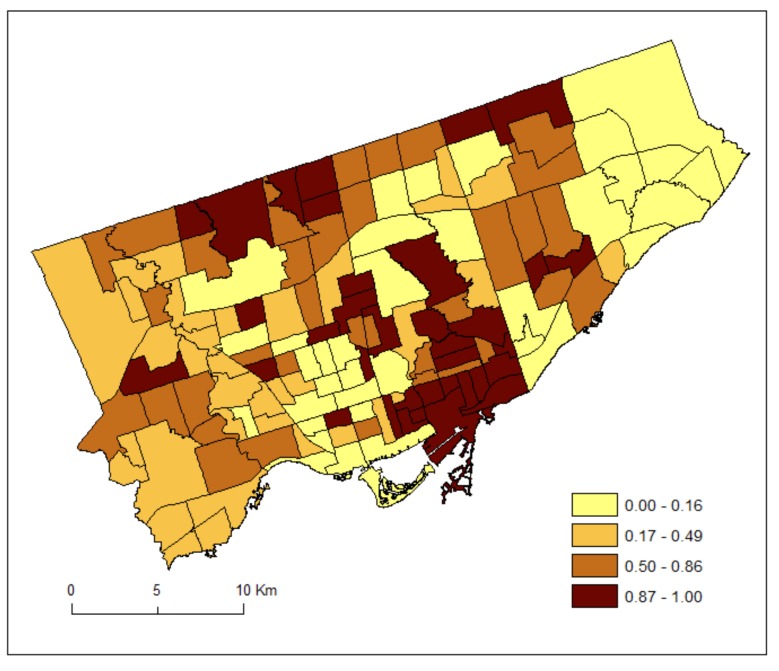
A quantile map of the posterior probabilities that the true underlying risk of MH service use was greater than one.

The (latent) shared component (θ) is a spatial process shared by both DV and HA that represents the neighbourhood-level spatially structured variability of MH risk common to the two data sources of DV and HA on MH. It can be interpreted as the true underlying risk surface of MH that are based on both DV and HA in the study region. Figure 5 maps this true risk surface of MH, i.e., posterior medians of exp(θ_i_), and Figure 6 maps the posterior probabilities that the true underlying risk were greater than 1. These maps help to identify neighbourhoods where risks of DV and HA were similar.

The posterior median of the scaling parameter (δ) equals 0.48 (95% CI: 0.35, 0.71; CI denotes credible interval). We also calculated the fraction of total variation in relative risks for DV or HA that is explained by the shared component. They were 0.45 (95% CI: 0.17, 0.81) and 0.66 (95% CI: 0.29, 0.94), respectively. We discuss below the results. 

## 4. Discussion

Based on treated or recorded MH conditions, the approach of SCSM enables estimation of additional information on the common (DV and HA) and discrepant (DV or HA) geographic patterns of MH conditions related services through borrowing strength across services as well as across neighbouring areas. Figure 3 shows that risks of DV were higher in the southwestern part and midtown of Toronto and lower in the far eastern part. These hotspots may reflect relatively good supply of doctors, or that MH conditions were supported at the community level thus minimizing the needs of hospital services. Policy makers may want to check service records for wait times of doctor services in these neighbourhoods. Increasing community-based MH services in these neighbourhoods could be helpful as they had high utilization of DV compared to other neighbourhoods. To examine equity in access to care, future research should explore if neighbourhoods that have better physician access also have higher socioeconomic status.

Contrastingly to Figure 3, Figure 4 shows that utilizations for HA were higher in the eastern part (but low for DV), and lower in the southwestern part (but high for DV). Neighbourhoods with high HA but low DV (eastern part) may suggest a lack of doctor supply or limited accessibility to doctors, requiring long travelling time, for instance. This finding may suggest that a lack of physician visits results in greater utilization of hospitals for MH reasons. However, it may also be that there is also a lack of community-based MH services, such as case management or assertive community treatment. This analysis, however, does indicate a need to review the supply and accessibility of physician services for supporting persons with MH conditions in areas where HV is high. Additionally, there may be a need to promote earlier intervention both in terms of public awareness of the early signs of mental illness and where to seek help as well as increasing the supply of primary care services that can provide early intervention.

We have used the shared component (θ) to estimate the common risks for DV and HA, and the scaling parameter (δ) to estimate the difference between the magnitudes of their area-specific risks. As can be seen from Equations (7) and (8), a study region could have overall high risks of DV but low in HA and thus resulting in a high value of δ. Alternatively, a study region could have overall low risks of DV but high risks in HA and thus resulting in a low value of δ. If the risk gradients from DV and HA are similar, then δ should be close to 1. Our result of the posterior median of the scaling parameter (δ) equals 0.48. This implies that the ratio of the risk gradients (δ and 1/δ) of DV and HA equals 0.23, suggesting that the shared component was playing a more important role to the area-specific risks of HA than those of DV. Indeed, we can see from Figure 3 and Figure 4 that the highest risks for DV and HA were 1.16 and 1.76, respectively. 

We further analysed the shared component that may be indicative of the true (underlying) risk surface of MH from DV and HA. A comparison of the fraction of total variation in relative risks for DV and HA that was explained by the shared component suggests that the shared component could better explain the variation of HA. Over half (66%) of geographic variation of HAs was recognized by DVs as well, but less than half (45%) of geographic variation of DVs were also recognized by HAs. This sounds reasonable as utilization of hospital services in general is less complicated to identify or record correctly than doctor visits. 

Results of the shared component that identify true risks enable the prioritization of specific neighbourhoods for implementing intervention strategies. Figure 5 maps the true underlying risk from DV and HA and Figure 6 maps the probability of the risk greater than one. The darkest areas on the map in Figure 5 showed the neighbourhoods with relatively higher risk of mental health service use. Additionally, we can see from Figure 6 that neighbourhoods with higher true underlying risks greater than one were located in the southern part around the downtown areas. The darkest areas on the map in Figure 6 represent neighbourhoods that had the highest probability of risk greater than one. For these neighbourhoods, the demand for MH services was particularly high. In these areas, a review of wait times for physician services or repeat emergency department or hospital visits may be useful to assess if both doctor and hospital services for MH should be increased, in addition to the supply of community-based MH services. In addition to equitable support within neighbourhoods (e.g., supported housing, income support), there is a need to consider equitable prevention. This involves analysis of factors such as socio-environmental marginalization within neighbourhoods, in addition to individual factors, in relation to MH status and health service, including trends over time. Such analyses framed using the behavioral model of health service use can identify inequities in MH outcomes and access to care. It should also be useful to consider integrated knowledge exchange strategies to develop a policy learning community related to MH.

Our results support action to reduce health inequities in Toronto, focusing on MH. Assuming that there were no socio-economic factors that had resulted in a geographic variation in risk, if health inequalities in terms of service provision did not exist, then the risk to DV and HA should be similar in all neighbourhoods. Additionally, if they are not similar as shown in our findings, then health inequalities could be playing an important role in affecting MH, including access to community-based physician care. The supply of psychiatrists varies considerably across Ontario. Kurdyak et al. [7] found inequities in the supply and visit patterns of psychiatrists across Ontario. The supply was highest in the Toronto Central region of Ontario with 62.7 per 100,000 but these psychiatrists had smaller panels of patients and fewer overall visits per year. Interestingly, within regions there was also variability in the characteristics of patients who received more outpatient care. In Toronto, patients who had more than 16 visits per year were less likely to have been admitted to inpatient psychiatry in the prior two years and reside in areas of higher income compared to patients with less than four visits per year. Health system inequities could be playing an important role in affecting MH, including access to community-based physician care.

By analyzing simultaneously the use of DV and HA services, we obtained a risk surface of MH specific for DV or HA, which may suggest different risk factors for each one, and a risk surface common to both DV and HA, which may suggest common risk factors. If people (e.g., age groups, ethnicity), environment (e.g., healthy/unhealthy food, walkability) and health services (e.g., easy access to doctors) are similar in each neighbourhood, then the risks of DV and HA due to MH across neighbourhoods should be more similar than what were shown on our maps of results. If people across neighbourhoods were similar, then the environment or/and health services across neighbourhoods must be different in order to account for the difference in risks. 

For this study, we have focused our discussions on health services, so assuming that the people (e.g., socioeconomic characteristics) and the environment are the same. Adding covariates of risk factors to our model can inform on the extent to which the shared component is picking up on the covariate(s). For example, if the shared component is flattened out when deprivation, a possible cause of inequity, is added to the model, then deprivation could be part of the missing information that is shared by both DV and HA. Several studies have identified contextual factors associated with health service use. For instance, residing in areas of high material deprivation predicts greater use of primary and hospital-based mental health services [5]. Johnson et al. [29] found that the supply of publicly funded mental health services in different regions of Virginia, U.S.A was not consistent with need, based on the estimated prevalence of mental health conditions per region. Future research should explore risk factors associated with the shared or individual risks as the different geographical patterns between DV and HA suggests specific features of DV and HA regarding MH, and the true underlying risks of MH displayed on the shared component map clearly suggests hotspots that could likely be explained by geographic variation of socioeconomic factors and MH services. 

In this study, SCSM has provided a useful approximation to the underlying risk surface of MH, as noted by Knorr-Held and Best [13]. It has also shed light to the pattern of exposures that is relevant to DV and HA. Adding covariates to the model could provide insight into potential risk factors. However, the random effects in the model that only has relevance to the kth outcome are assumed pair-wise independent, which means that the model does not allow for interaction between covariates. A study that compared SCSM with or without covariates noted that SCSM with covariates needs to be carefully explored by considering possible pair-wise dependence with respect to the residual [25]. Such consideration in future research should be helpful for identifying risk factors and estimating a more accurate underlying risk surface of MH from multiple outcomes. 

There were data limitations in this study. The data for hospital admissions were estimated from counts during two years (1 April 2012–31 March 2014) and 2011 Census population estimates. The data for DV and HA cover different observation periods, 2011–2012 and 2012–2013, respectively. For our DV data, we are unsure how much they represent Primary Care since we do not know if the billings were for a primary care visit, or psychiatry or other specialist consultation. Since we were using data that were already aggregated neighbourhood level, we were not able to identify DVs that occurred in outpatient settings from DVs that occurred in relation to a hospital admission. In Ontario there are instances where physicians may be salaried staff of the hospital while in other instances physicians have practicing privileges at a hospital and bill OHIP for services rendered. The latter would influence our indicator of DVs. Therefore, our results might be illustrating DVs associated with a hospital admission in some areas of shared risk. This does not necessarily change the possibility that individuals with greater MH need may reside in these areas or that there are disparities in appropriate outpatient care in these areas given. However, future research using data that represents only DVs occurring as outpatients will provide greater insights into the patterns MH service use across neighbourhoods in Toronto.

## 5. Conclusions

The study aims to provide evidence from DV and HA to inform and improve public health policy and practice on MH. It has identified variability in the use of DVs and HAs across areas in Toronto, with some areas showing high use of both. There may be gaps in community resources, including primary care, in areas with high hospital visits. Assuming that there were no biases in data collection of DV and HA, hotspots may reflect dissimilarities of risk surfaces due to differences (or inequalities) in the availability of primary care (doctor) services and/or differences in the disease stage targeted by the data sources. For instance, areas high in DV may have a population of persons with MH conditions that are more easily managed in the community (e.g., depression) compared to areas higher in HA where more persons with conditions that are really brain disorders and may not be preventable such as schizophrenia or bipolar disorder may reside. On the other hand, hotspots may indicate disparities in the availability or access to outpatient MH services. This would be consistent with findings from Kurdyak et al. [7] that showed wide variations in the availability of outpatient and inpatient psychiatry services. There may be greater risk of HAs when there is less access to primary care, in areas further away from hospitals with MH beds and/or greater numbers of individuals with more advanced illness in areas closer to hospitals. Finally, the finding that in some areas risk for DV was high and low for HA might suggests that hospitalizations are potentially preventable when primary care services are sufficient or better planned. Further research is needed to explain the different geographic patterns identified.

The methodology of SCSM shows promise for use in health system planning and evaluation. Using a local spatial analysis enables specific areas to be identified for improving health inequalities. Further, using a spatial shared component modeling approach enables different components to be combined, analyzed and compared spatially in one model. Visits to DV and HA due to MH may share common risk factors such as deprivation. The fact that neighbourhoods with common risks of DV and HA exist, highlighted by the shared component model, provides convincing evidence of real clustering in the underlying risk surface of MH in Toronto. Future research should aim to explore reasons behind the geographic variation of utilization of DV and HA. Factors like locations and counts of doctors and hospitals might have played some important roles. Efforts should also be made to acquire and use more specific data of service utilization: primary care visit, psychiatry, or other specialist consultation et cetera. It would also be useful to study specific age groups to investigate how accessibility to physicians and other primary health care services could be affecting risks of MH for young or old adults, for instance, in different neighbourhoods. Using SCSM that contains (actual) covariates with careful considerations of their interactions could help to identify common risk factors of DV and HA due to MH at the neighbourhood level and also separate risk factors that are specific to DA or HA. For instance, we could add accessibility to doctors based on their office locations as a covariate to see if areas with high DV risk diminish after controlling for doctor accessibility. We recommend a more in-depth study on how intervention could be implemented at the provincial, municipality, city, and neighbourhood level to improve primary health care services and health inequalities, which could help to reduce prevalence of MH.
